# Transcriptome Analysis of Capsicum Chlorosis Virus-Induced Hypersensitive Resistance Response in Bell Capsicum

**DOI:** 10.1371/journal.pone.0159085

**Published:** 2016-07-11

**Authors:** Shirani M. K. Widana Gamage, Desmond J. McGrath, Denis M. Persley, Ralf G. Dietzgen

**Affiliations:** 1 Queensland Alliance for Agriculture and Food Innovation, The University of Queensland, St Lucia, Queensland, Australia; 2 Queensland Department of Agriculture and Fisheries, AgriScience Queensland, Gatton, Queensland, Australia; 3 Queensland Department of Agriculture and Fisheries, AgriScience Queensland, EcoSciences Precinct, Dutton Park, Queensland, Australia; Washington State University, UNITED STATES

## Abstract

**Background:**

Capsicum chlorosis virus (CaCV) is an emerging pathogen of capsicum, tomato and peanut crops in Australia and South-East Asia. Commercial capsicum cultivars with CaCV resistance are not yet available, but CaCV resistance identified in *Capsicum chinense* is being introgressed into commercial Bell capsicum. However, our knowledge of the molecular mechanisms leading to the resistance response to CaCV infection is limited. Therefore, transcriptome and expression profiling data provide an important resource to better understand CaCV resistance mechanisms.

**Methodology/Principal Findings:**

We assembled capsicum transcriptomes and analysed gene expression using Illumina HiSeq platform combined with a tag-based digital gene expression system. Total RNA extracted from CaCV/mock inoculated CaCV resistant (R) and susceptible (S) capsicum at the time point when R line showed a strong hypersensitive response to CaCV infection was used in transcriptome assembly. Gene expression profiles of R and S capsicum in CaCV- and buffer-inoculated conditions were compared. None of the genes were differentially expressed (DE) between R and S cultivars when mock-inoculated, while 2484 genes were DE when inoculated with CaCV. Functional classification revealed that the most highly up-regulated DE genes in R capsicum included pathogenesis-related genes, cell death-associated genes, genes associated with hormone-mediated signalling pathways and genes encoding enzymes involved in synthesis of defense-related secondary metabolites. We selected 15 genes to confirm DE expression levels by real-time quantitative PCR.

**Conclusion/Significance:**

DE transcript profiling data provided comprehensive gene expression information to gain an understanding of the underlying CaCV resistance mechanisms. Further, we identified candidate CaCV resistance genes in the CaCV-resistant *C*. *annuum x C*. *chinense* breeding line. This knowledge will be useful in future for fine mapping of the CaCV resistance locus and potential genetic engineering of resistance into CaCV-susceptible crops.

## Introduction

Capsicum (*Capsicum* spp.) is a genus in Solanaceae, a family that contains economically important crops including potato and tomato. Capsicum is native to Central and South America comprising 30 species but only 5 species, *C*. *annuum* L., *C*. *baccatum* L., *C*. *chinense* Jacq., *C*. *frutescens* L., and *C*. *pubescens* have been domesticated [1]. *Capsicum* spp. are grown worldwide for their nutritional and condimental value. Pungent capsicum, such as paprika, chilies and hot pepper are used as spices and sweet capsicum such as Bell capsicum is consumed as vegetable. In 2012, world fresh capsicum production reached 31 million tons with highest contribution from China (www.fao.org). As with other crops, capsicum is attacked by various pathogens and pests that can cause extensive losses in production. Pathogens, such as *Xanthomonas* spp., *Phytophthora* spp, *Colletotrichum* spp., chili leaf curl virus, tomato spotted wilt virus (TSWV) and groundnut bud necrosis virus (GBNV) have been reported to cause major diseases in capsicum [2–4]. Recently, capsicum chlorosis virus (CaCV) emerged as a serious pathogen of capsicum and chili in Australia and India, respectively [5, 6]. In addition, CaCV infects tomato and peanut crops in Australia, Thailand and China [5, 7, 8]. Disease symptoms on capsicum leaves include marginal and interveinal chlorosis with narrow strap-like appearance, mottling, ring spots, chlorotic spots and tissue necrosis on the top leaves while infected capsicum fruits often become distorted with necrotic lesions [5, 9, 10]. CaCV is known to be transmitted by three species of thrips, *Ceratothripoides claratris*, *Frankliniella schultzei* and *Thrips palmi* in a circulative and propagative manner [5, 7]. Taxonomically, CaCV is a tentative species in the genus *Tospovirus*, family *Bunyaviridae* [11]. It contains a tripartite single strand RNA genome with negative and ambisense polarity which codes for RNA-dependent RNA polymerase, two structural proteins, nucleocapsid and glycoproteins and two non-structural proteins with proposed suppressor of RNA silencing and movement protein functions [12]. CaCV protein that triggers susceptibility or resistance responses in host plants remains unresolved.

Commercial cultivars carrying CaCV resistance are presently unavailable. As a result, breeding programmes’ search for natural CaCV resistance has become increasingly urgent. In Australia, CaCV resistance has been identified in several *C*. *chinense* Plant Introduction (PI) accessions. A sub-line obtained through self-pollination of *C*. *chinense* PI290972 showed uniform resistance to field isolates of both TSWV and CaCV [5]. Previously, TSWV resistance (*Tsw*) had been identified in *C*. *chinense* PI 152225 and PI 159236 [13]. However, *Tsw* resistance is not effective against CaCV [11]. TSWV resistance identified in *C*. *chinense* PI290972 appeared to be due to *Tsw* since resistance was overcome by a *Tsw* resistance-breaking strain [5]. CaCV resistance in *C*. *chinense* PI290972 is thought to be governed by a single dominant gene that segregates independently from TSWV resistance (DJ McGrath and DM Persley, unpublished). A current breeding program by the Queensland Department of Agriculture and Fisheries (DAF) transferred CaCV resistance identified in PI 290972 into commercial Bell capsicum cultivars. Genomes of *C*. *annuum* and *C*. *chinense* PI159236 [14, 15] were recently sequenced. These genomes laid the foundation for further studies on genetics and genomics combined with Next Generation Sequencing (NGS) including RNA-Seq. RNA-Seq provides a powerful tool to identify differential expression of genes [16] and has been previously used to investigate the molecular basis of host plant responses to pathogens including viruses [17–22]. The objective of the present study is to investigate molecular responses of resistant Bell capsicum line to CaCV infection and to identify candidate CaCV resistance genes. We used comparative transcriptome profiling of CaCV-susceptible and -resistant Bell capsicum cultivars using Illumina NGS platform to compare transcriptional changes in response to CaCV infection. Detailed analysis of the most highly up-regulated genes in the resistant line revealed at least 45 annotated genes associated with plant defense response at different levels, such as localized cell death, cell signalling and synthesis of defense-related secondary metabolites. Furthermore, we identified candidate CaCV resistance genes and predicted their chromosomal location.

## Materials and Methods

### CaCV inoculation and RNA extraction

Bell capsicum (*Capsicum annuum* L.) seedlings from a CaCV-resistant (R) and a CaCV-susceptible (S) line were grown at 25°C and 16 h light/8 h dark conditions. CaCV-resistant line was derived from the DAF breeding program aimed to introgress CaCV resistance from *C*. *chinense* (PI 290972) into commercial Bell capsicum. The R seedlings used were from the third backcross generation of PI 290972 x *C*. *annuum* cv. Mazurka and cv. Warlock inbred lines. Commercial cultivar *C*. *annuum* cv. Yolo Wonder, an open pollinated inbred line, was used as CaCV-susceptible line. Seedlings aged 4–5 weeks were mechanically inoculated with CaCV isolate QLD 3432 [23]. Inoculum was prepared by grinding fresh CaCV-infected symptomatic capsicum leaves of cv. Yolo Wonder in 10 mM phosphate buffer, pH 7.6 with freshly added 20 mM sodium sulphite. Inoculum was rub-inoculated onto Carborundum-dusted leaves. Control plants were rub-inoculated with buffer (mock). Inoculated leaves were harvested from all treatments at the time point when CaCV-challenged R capsicum plants had developed necrotic lesions, which for this experiment was 4 days post inoculation (dpi) and were stored at -80°C until RNA extraction. To minimize biological variability, total RNA was extracted from three biological replicates for each treatment that consisted of at least 3 plants each. Briefly, pools of leaves of at least 3 plants were ground in liquid nitrogen and RNA was extracted from 100 mg of leaf powder using an RNeasy Plant Mini Kit (Qiagen) following the manufacturer’s protocol. RNA was eluted in 30 μl of RNase-free water. RNA preparations were treated with DNase to remove genomic DNA using a Turbo DNA-free kit (Ambion) following the manufacturer’s protocol. Presence or absence of CaCV in RNA preparations was confirmed by RT-PCR using CaCV N gene-specific primers forward 5’-ATGTCTAACGTCAGGCAACTT-3’ and reverse 5’-CACTTCTATAGAAGTACTAGG-3’.

### Illumina sequencing and bioinformatics

Illumina sequencing, quality control, alignment, transcript assembly, quantification, normalization and expression profiling comparisons were outsourced to the Australian Genome Research Facility (AGRF, Melbourne, Australia). Briefly, rRNA in total RNA preparations was depleted as the first step to enrich non-rRNA species including pre-mature mRNA and long non-coding RNA using Ribo-Zero Plant rRNA Removal Kit (Epicentre, Illumina). Next, RNA quality and concentration were determined prior to library preparation using Agilent 2100 Bioanalyzer (Agilent Technologies). In total 12 cDNA libraries were prepared using poly (A) enrichment from 3 replicates of 4 treatments consisting of samples from CaCV- or mock-inoculated R and S lines. One lane of an Illumina HiSeq 2000 sequencer was used to generate 100-bp paired-end reads. High quality reads obtained through FastQC filter were further cleaned to remove sequences of cross species contamination including CaCV [23]. Clean reads were aligned against *C*. *annuum* L. Zunla-1 complete genome [15] using Tophat aligner [16]. Transcript abundance and differential gene expression were calculated using Cufflinks with reference based assembly option (RABT) which generates known and potentially novel transcripts [16]. Differential expression of genes was compared between R and S lines using CaCV- and mock-inoculated conditions. Cufflinks generated normalized read counts per gene per sample with fragments per kilobase of exon per million mapped reads (FPKM) values. Significantly differentially expressed genes were determined by using a binary statistical assessment. Briefly, a p-value was calculated for each gene in each sample and comparison. Then p-values were corrected for multiple tests and comparisons (q-value) using false discovery rate (FDR). In this experiment, a FDR 0.05 cut off was used to determine p-value threshold.

### Functional annotation of differentially expressed genes

We applied stringent filtering criteria to select highly significant differentially expressed genes (DE) at least two-fold between two conditions by setting cut off q-value < 0.01 and log2 fold change > 1. Selected DE genes were classified into functional categories using Blast2GO with default parameters [24]. Annotations were fine-tuned by using Annex-based gene ontology (GO) term augmentation followed by removal of First Level GO terms and mapped to GOSlim (plant). KEGG enzyme codes were assigned for annotations using Blast2GO to identify which biological pathways are catalyzed by DE enzymes. Enzyme codes were mapped into KEGG database [25].

### Validation of DE genes by real-time quantitative PCR

RNA-Seq gene expression profiles for 15 selected genes were validated using real-time quantitative PCR (qPCR). Four genes, actin, TATA box-binding, nuclear cap-binding and galactono-lacton dehydrogenase (GLDH), were selected from non-DE genes in the RNA-Seq dataset as internal reference genes. Primers for target and reference genes were designed using Primer3 [26, 27]. Primer sequences are listed in S1 Table. Complementary DNA was synthesized with oligo dT primers using Superscript III First-strand cDNA synthesis kit (Life Technologies) using the same total RNA preparations (DNase-treated) used for Illumina sequencing. SensiFAST SYBR No-ROX Kit (Bioline) was used in a Rotor-Gene Q real-time PCR cycler (Qiagen) with 20 μl of final volumes containing 1 μl (20 ng) cDNA, 0.8 μl of each primer (10 μM), 7.4 μl of DNase- and RNase-free water and 10 μl of 2x SYBR No-ROX mix. Reaction conditions were 2 min at 95°C followed by 40 cycles of 95°C for 5s, 60°C for 10s and 72°C for 20s. Experimental design and subsequent data analysis methods applied were in accordance with a previously described protocol [28]. Three biological replicates and two technical replicates were used per sample. Since all reference genes showed stable gene expression across all samples and treatments, we arbitrarily chose actin for all future experiments. Real-time PCR efficiencies of target genes and actin were determined by standard curve method using a ten-fold dilution series of cDNA (from 100 ng to 0.001 ng). PCR efficiencies were calculated from the slopes of standard curves. Threshold cycle (Ct) number was determined from log scale amplification curves. Reaction efficiencies for target and reference genes showed 95–100% efficiency for 1–100 ng of cDNA template input. Hence, it was decided to use 20 ng of cDNA template for further reactions. For each reaction, no-template and no-RT control samples were included. Relative expression levels of target genes were calculated as 2^−(*Ct of target−Ct of reference*)^ [29]. Fold changes in gene expression between treatment and control were calculated using 2^−ΔΔ*Ct*^ method; 2^−(Δ*Ct of treatment*−Δ*Ct of control*)^ [29]. For validation, qPCR derived log2-fold changes were compared with log2-fold values obtained through RNA-Seq analysis.

## Results and Discussion

### Transcriptome sequencing and identification of differentially expressed genes

To determine genome-wide CaCV resistance responses in *C*. *annuum*, we sequenced and compared transcriptome of a CaCV-resistant breeding line (R) and a susceptible (S) commercial cultivar using Illumina next generation sequencing. This is the first such analysis for CaCV molecular interactions with capsicum. Previously, molecular responses of host plants to impatiens necrotic spot virus (INSV) and TSWV tospovirus infections were reported using microarrays derived from expressed sequence tags. In *N*. *benthamiana*, INSV infection led to the up-regulation of genes involved in plant defense, transcription, signal transduction, protein synthesis and metabolism in comparison to mock plants while expression of histones was noticeably down-regulated [30]. In chrysanthemum, TSWV infection led to the up-regulation of chitin response genes and down-regulation of genes involved in DNA replication, chromatin biogenesis and cytokinesis compared to mock plants [31]. However, these studies targeted molecular responses to tospovirus infections in susceptible host plants. This report is the first study to determine molecular host plant resistance responses to infection by a tospovirus.

**Fig 1:**
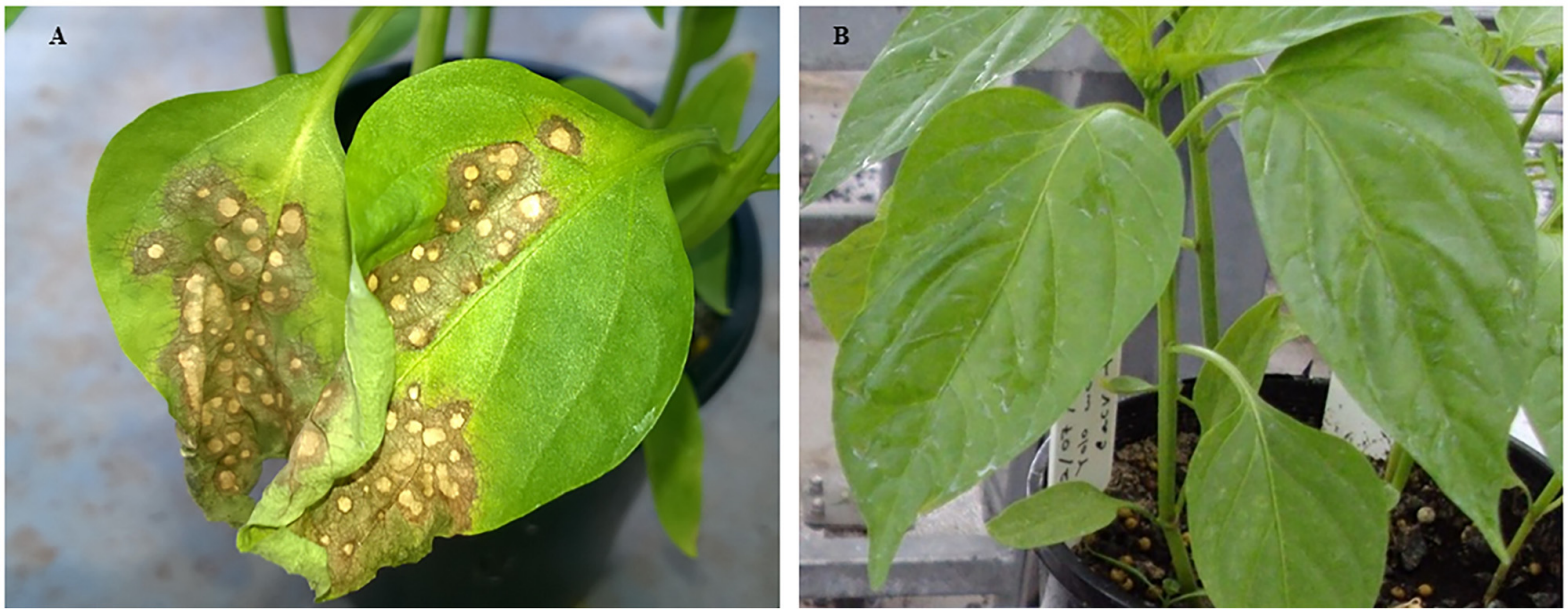
Symptoms on inoculated leaves of *C*. *annuum* following CaCV infection 4 days after mechanical inoculation. CaCV-resistant line showing coalescing necrotic lesions (A) and susceptible line without visible symptoms (B).

We compared the transcriptome response of CaCV-resistant and susceptible capsicum in the presence and absence of CaCV infection. Due to a lack of CaCV resistance in commercial cultivars, in this study we used a resistant backcross line from our breeding program. R and S lines were mechanically inoculated with a Queensland isolate of CaCV, whereas control plants were mock-inoculated with buffer. When challenged with CaCV, plants carrying CaCV resistance gene showed strong hypersensitive response (HR) on inoculated leaves with necrotic lesions and premature leaf abscission at 4 dpi while susceptible plants did not show any symptom at this early time point (Fig 1). We extracted total RNA from symptomatic inoculated leaves at 4 dpi to determine resistance response against CaCV. As controls, total RNA was extracted from CaCV-inoculated leaves of S line and mock-inoculated R and S line plants at the same time point. RT-PCR of total RNA extracts using CaCV N gene-specific primers confirmed that all CaCV-inoculated plants were indeed infected and mock-inoculated plants did not contain any detectable CaCV. Illumina HiSeq generated 79,562,608 paired-end (100-bp) clean reads distributed across 12 cDNA libraries. Sequence reads were mapped against complete genome of a Chinese inbred line *C*. *annuum* L. Zunla-1. In total 32,939,994 (41.4%) reads mapped to this reference genome with 10–30 minimum to maximum exon coverage. This unexpectedly low percentage may be due to the fact that for this analysis only unique, paired reads were counted and the available reference genome is only a draft assembly that still contains gaps.

Reads that mapped to genes of the capsicum reference genome were calculated in two pairwise comparisons; R/CaCV vs S/CaCV and R/mock vs S/mock. Mapping reads to the reference genome identified 37,502 and 38,417 transcripts for R/CaCV vs S/CaCV and R/mock vs S/mock comparisons, respectively. Of these transcripts, 34,905 (93.1%) and 34,986 (91.1%) transcripts, respectively, corresponded to predicted protein coding regions (> 300bp) in the reference capsicum genome. Excluding transcripts smaller than 300 bp, 2270 and 3035 transcripts in R/CaCV vs S/CaCV and R/mock vs S/mock comparisons respectively could not be linked to protein coding regions in the reference genome. These transcripts are likely either non-coding RNAs or transcriptionally active regions in Zunla-1 genome which were not annotated during reference genome assembly or transcripts of genes that may have been introduced to Bell capsicum from *C*. *chinense* PI line during introgression. Bell capsicum RNA-seq transcriptome contained 34,905–34,986 protein-coding regions, similar to those of other capsicum genomes and transcriptomes suggesting sufficient coverage (S2 Table).

Normalized read counts (FPKM values) between two samples were statistically compared to determine whether a gene was significantly differentially expressed (DE) or not. A gene was considered significantly DE when the q-value was <0.01 and log2-fold >1.0. None of the genes in mock-inoculated plants were DE between S and R lines, indicating a common response to rub-inoculation treatment. In contrast, when R and S lines were CaCV-inoculated, 2484 DE genes were identified. Hence, we assumed that these genes were DE in response to CaCV infection.

### Functional classification of DE genes in response to CaCV

**Fig 2:**
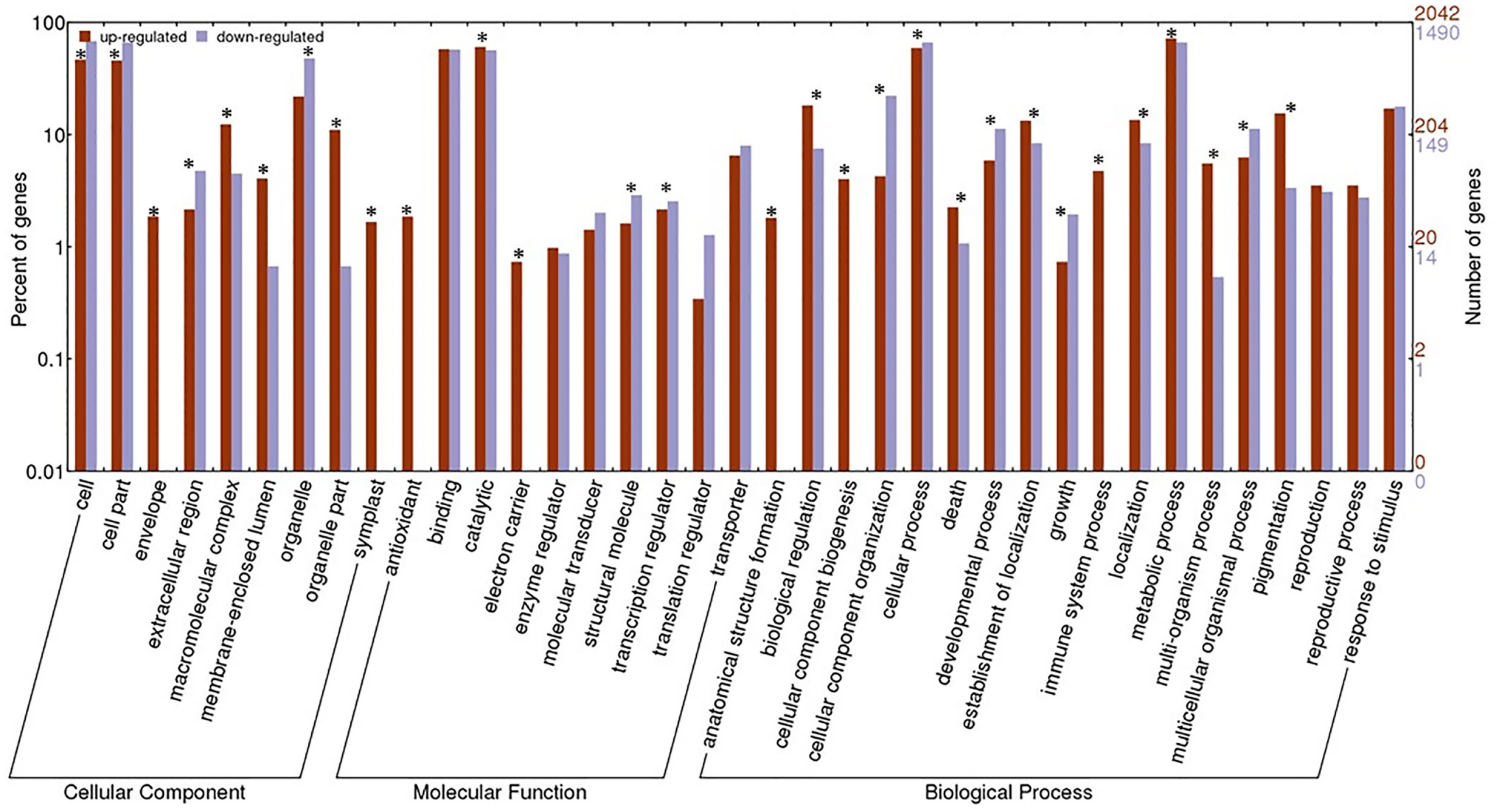
Gene ontology categories of differentially expressed genes in *C*. *annuum* in response to CaCV infection. The number and percentage of up- or down-regulated genes in CaCV resistant line classified into three major domains were plotted using WEGO tool. Significant relationship (p< 0.05) between up- and down-regulated genes indicated with ‘*’.

To determine putative functions of DE genes that appear to be involved in resistance responses against CaCV infection, DE genes were subjected to gene ontology (GO) analysis using Blast2GO software [24]. Of the 2484 DE genes, 1316 (53%) were found to be up-regulated in R line, while 1168 (47%) were down-regulated. Only 273 (11%) genes were uniquely expressed in R line and 70 (2.8%) in S line. Such uniquely expressed genes may play a critical role in plant resistance and susceptibility to CaCV. However, expression of majority of genes in common in R and S lines, but at different levels, suggests resistance and susceptibility to CaCV is underpinned by broad up-regulated general defence responses rather than expression of different sets of genes. Up- or down-regulated genes were separately classified into functional categories. In total 2603 up-regulated and 1754 down-regulated transcript sequences were uploaded into Blast2GO. Transcript counts were higher than corresponding gene counts due to the presence of transcript isoforms, which may arise due to alternative splicing events in multi-exon genes [32]. Blast2GO annotated 2042 (78.4%) up-regulated transcripts that code for 1019 predicted protein-coding genes and 1490 (84.9%) down-regulated transcripts that code for 766 predicted-protein coding genes. DE genes were classified into 3 major functional domains, Biological Process (BP), Molecular Function (MF) and Cellular Component (CC). GO assignments in up-regulated genes included MF; 571, BP; 310 and CC; 139, and in down-regulated genes BP; 334, MF; 323 and CC; 127. GO annotation results were plotted using Web Gene Ontology (WEGO) Annotation tool to compare proportions of up- and down-regulated GO terms (Fig 2) [33].

Within BP domain, a large number of DE genes were placed in the categories of ‘metabolic process’ (80.2%) and ‘cellular process’ (71.6%) irrespective of being up-or down-regulated. Genes involved in ‘immune system process’, ‘cellular component biogenesis’ and ‘anatomical structure formation’ were found restricted to up-regulated genes or in other words specific to R line response. Within MF domain, a large number of DE genes fell into ‘binding’ (66.4%) and ‘catalytic activity’ (67.8%). Genes involved in ‘electron carrier activity’ and ‘antioxidant activity’ were uniquely expressed in R line. Within CC domain, majority of DE genes were found to be involved in producing proteins localized in ‘cell’, ‘cell part’ and ‘organelle’. Proteins that are localized in ‘symplast’ and ‘envelope’, including cellular membrane associated protein genes were found to be R line specific.

Assuming that genes that were up-regulated in R line are linked to disease resistance, functional annotations were searched for GO categories that showed direct or indirect links with plant resistance response and compared with known functions of homologues. Previously reported transcriptional responses to viral infection in resistant host plants showed up-regulation or overexpression of genes assigned to several GO categories. For instance, in tomato, genes related to defense response, cell wall reorganization, transcriptional regulation and secondary metabolite synthesis were up-regulated in response to tomato yellow leaf curl virus [17]. In Cassava, genes related to hormone signalling and synthesis of secondary metabolites were overexpressed in response to ipomovirus infection [18]. During an early infection of potato Y virus in potato, transcripts classified in GO terms ‘photosynthesis’, ‘light harvesting’, ‘protein-chromophore linkage’, ‘response to auxin stimulus’ and ‘negative regulation of peptidase activity’ were specifically up-regulated [19]. In this report, we specifically focus on the most highly up-regulated genes with GO terms, ‘defense response’, response to stress’, ‘signal transduction’ and GOs related to hormone mediated signalling and secondary metabolism. Potential roles of such genes during capsicum-CaCV hypersensitive defense response are discussed below. Further, putative disease resistance genes identified in the dataset are also discussed.

### Defense-related genes

GO analysis showed 60 genes were associated with ‘defense response’ category and 15 genes with ‘response to stress’ (S3 Table). Of these groups of genes, 16 were among the most highly up-regulated (log2-fold > 3.0, FPKM >300) 45 genes listed in Table 1 and include genes, such as those coding for pathogenesis-related (PR) proteins. PR proteins are known to be induced upon pathogen invasion and restrict pathogen development and spread [34]. The most highly up-regulated PR protein gene was PR leaf protein 4-like, which showed close relation to PR-1 type proteins. Interestingly, the most highly up-regulated R line specific (only expressed in R line) PR protein gene was also a precursor of PR-1. Although PR-1 represents a highly conserved family of plant proteins, mechanism by which PR-1 proteins affect pathogens remain to be elucidated [34, 35]. According to Blast2GO analysis, GO assignments for PR leaf protein 4-like and PR-1 precursor proteins were ‘defense response’ and ‘extracellular region’. These GO assignments and higher expression levels are tempting to speculate that PR-1 type proteins of capsicum may play specific roles in defense against CaCV. Defensin-like PR proteins were also identified among the up-regulated genes. Plant defensins are known to possess broad antimicrobial activities and are induced by ethylene [34, 36, 37]. Another class of PR proteins induced in capsicum transcriptome was thionin-like proteins which are classified into PR-13 family [34]. Thionins are toxic secondary metabolites that act against fungi, bacteria and insects through membrane degradation [38, 39]. However, their role in plant-virus interactions remains unknown. It is likely that significant higher expression of thionins in this plant-pathogen interaction may contribute to the establishment of systemic acquired resistance [40]. In addition to PR-1 precursor, osmotin-like PR-5 protein was found specially expressed in R line. Other preferentially highly expressed PR protein genes in capsicum transcriptome include those encoding class II chitinase (PR-3) and lignin-forming anionic peroxidase (PR-9). We identified a highly expressed germin-like protein (GLP) gene that shared 99% identity to a previously characterized GLP1 in hot pepper (*C*. *annuum* cv. Bugang). Hot pepper GLP1 (*CaGLP1*) has been classified into PR-16 family and is thought to be involved in defense against viral and bacterial pathogens [41]. *CaGLP1* plant defense effect is assumed to occur through cell wall remodeling. Other GLPs function as enzymes, structural proteins and receptors [42]. The GLP in our dataset was classified in the ‘extracellular region’ suggesting localization outside cell wall. Accordingly, we speculate that this GLP may not be a receptor, but may function similar to *CaGLP1* that is involved in cell wall remodeling or possess an enzymatic activity. Significant up-regulation of PR family proteins appears to play important roles in capsicum defense against CaCV.

**Table 1:** List of most highly up-regulated DE (log2-fold > 3.0, FPKM >300) putative defense-related genes in CaCV-resistant *C*. *annuum x C*. *chinense* in comparison to CaCV-susceptible *C*. *annuum*.

Transcript Seq. ID	Seq. description (E-value)	FPKM (R line)	FPKM (S line)	log2- fold	GO term	Function
**22352**	Pepper esterase (0.0E0)	2132.7	3.0	9.46	Hydrolase activity	Defense [67]
**50328**	5-epi-aristolochene synthase (0.0E0)	1465.9	2.3	9.33	Defense response	Defense [43]
**30661**	Lipoxygenase (0.0E0)	1187.8	1.9	9.22	Biosynthetic process	Defense and cell death [44]
**39531**	PR leaf protein-4-like (6.3E-112)	3045.1	5.8	9.02	Defense response	Defense [34]
**03349**	1- aminocyclopropane-1-carboxylate oxidase (ACC oxidase) (0.0E0)	1760.4	4.3	8.67	Defense response	Defense [45, 46]
**19897**	Cytochrome P450 (0.0E0)	777.6	2.7	8.15	Oxidation and reduction	Defense [47]
**42568**	Apoplastic invertase (0.0E0)	434.9	1.6	8.09	Metabolic process	Defense [48]
**11987**	Cannabidiolic acid synthase-like (0.0E0)	938.5	3.6	8.03	Oxidation and reduction	Cell death [49]
**35659**	Proteinase inhibitor psi-1.2-like (1.97E-22)	1612.9	7.4	7.77	Serine-type inhibitor activity	Defense [50]
**04833**	Defensin-like (3.3E-39)	5268.4	26.3	7.64	Defense response	Defense [34]
**03989**	1-aminocyclopropane-1-carboxylate oxidase (ACC oxidase) (0.0E0)	1894.2	12.0	7.30	Defense response	Defense [45, 46]
**39790**	Omega-6 fatty acid desaturase endoplasmic reticulum isozyme-2 (0.0E0)	937.3	6.0	7.28	Lipid metabolic process	Defense [51, 52]
**31825**	Thionin-like protein (1.1E-40)	7903.6	52.0	7.24	Defense response	Defense [53]
**21202**	Pleiotropic drug resistance protein (PDR) 1 (0.0E0)	438.9	3.7	6.88	ATP binding	Defense [54]
**16797**	Kunitz-like protease inhibitor partial (0.4E-77)	726.9	6.7	6.76	Endopeptidase inhibitor activity	Defense [55]
**11123**	Hydroxymethylglutaryl-synthase-like (0.0E0)	345.1	3.8	6.50	Biosynthetic process	Defense [56]
**12646**	Class II chitinase (2.9E-170)	1875.7	22.3	6.39	Defense response	Defense [34]
**37429**	Uncharacterized protein LOC102581412 (0.0E0)	1183.6	14.4	6.36	Defense response	
**08215**	UDP-glucosyltransferase 1 (0.0E0)	431.6	5.5	6.30	Transferase activity	Defense [57]
**09442**	Lignin-forming anionic peroxidase (0.0E0)	1403.7	20.4	6.10	Response to stress	Defense [34]
**49199**	Probable aminotransferase TAT2 (0.0E0)	643.9	11.5	5.81	Biosynthetic process	Defense [58]
**03028**	Peroxidase 21 (3.1E-74)	475.9	8.6	5.78	Defense response	Defense [59]
**15678**	Alpha-dioxygenase 1-like (0.0E0)	683.4	15.1	5.50	Response to stress	Defense [60]
**11195**	Hydroxylmethylglutaryl coenzyme A reductase 2 (0.0E0)	498.9	11.8	5.39	Biosynthetic process	Defense [61]
**01189**	Glutathione S-transferase (4.2E-135)	336.4	8.6	5.29	Auxin activated signalling pathway	Multiple functions [62]
**43744**	Trypsin inhibitor 1-like (1.2E-33)	1034.8	26.9	5.26	Response to wounding	Defense [63]
**22424**	Abscisic stress-ripening protein 2 (2.0E-25)	891.2	25.2	5.14	Response to stress	Defense [64]
**16174**	Stellacyanin-like protein (5.1E-69)	870.6	28.4	4.94	Electron transport	Defense [65]
**15700**	Formate dehydrogenase mitochondrial (0.0E0)	896.6	39.1	4.52	Oxidation-reduction process	Defense [66]
**37151**	Tyramine n-hydroxycinnamoyl transferase (1.4E-180)	305.9	14.1	4.43	Biosynthetic process	Response to stress [67]
**00644**	Defensin-like protein (5.4E-21)	842.4	41.3	4.35	Defense response	Defense [34]
**35935**	Germin-like protein (1.1E-145)	409.6	22.8	4.16	Extracellular region	Defense [41]
**04723**	Stress induced protein 11 (6.7E-64)	815.1	55.0	3.88	Response to stress	Response to stress accession AHI85716
**21689**	Ornithine decarboxylase (0.0E0)	631.0	45.1	3.80	Biosynthetic process	Defense [68]
**31824**	Thionin-like protein (1.5E-19)	660.3	47.7	3.78	Defense response	Defense [53]
**10398**	Citrate synthase (0.0E0)	316.7	24.9	3.66	Metabolic process	Interact with plant hormone signalling pathways [69]
**05414**	Glutathione S-transferase (1.6E-83)	309.8	28.8	3.42	Protein binding	Carrier protein [70]
**13113**	Patatin-like phospholipase 1 (0.0E0)	470.1	55.7	3.07	Hydrolase activity	Defense [71]
**34250**	Proteinase inhibitor psi-1.2-like (3.4E-16)	4029.9	0.0	-	Serine-type inhibitor activity	Defense [50]
**36252**	PR-1 precursor (6.3E-131)	1233.0	0.0	-	Extracellular region	Defense [34]
**00016**	Hydroxymethylglutaryl- synthase-like (7.1E-34)	957.0	0.0	-	Biosynthetic process	Defense [56]
**33576**	Non-symbiotic haemoglobin 1 (3.4E-87)	547.3	0.0	-	Oxygen binding	Defense [72]
**26713**	Cytochrome P450 cyp736a12-like (6.8E-129)	407.0	0.0	-	Oxidation-reduction process	Unknown
**04631**	Probable glutathione S-transferase (2.1E-137)	384.6	0.0	-	Protein binding	Carrier protein [70]
**05987**	Osmotin-like (3.2E-173)	368.4	0.0	-	Defense response	Defense [34]

Proteinase inhibitors (PIs) are another kind of defense-related proteins known to provide defense against insects and pathogens in plants that induce formation of abscission zone and thereby sacrifice tissues to prevent further invasion of pathogens [34, 73, 74]. We identified several highly up-regulated PI proteins including proteinase inhibitor PSI-1.2 [50]. PSI-1.2 was the most highly expressed R line-specific gene with 4030 FPKM. Proteinase inhibitor 1-B was another highly expressed PI, which was classified with ‘response to wounding’ GO term indicating direct involvement in stress response. Other than the above-mentioned PIs, a Kunitz-like protease inhibitor gene was expressed with 6.67 log2-fold change. Kunitz family PIs are involved in regulation of programmed cell death or apoptosis during HR of plant response to pathogens [75]. The role of these proteins in plant defense is further supported by a Kunitz family trypsin and protease inhibitor gene that was reported up-regulated in lethal systemic HR in soybean in response to soybean mosaic virus infection [20]. It is likely that the highly up-regulated PI protein genes, especially those specific for R line, are involved in HR and induction of premature leaf abscission of necrotic leaves that was observed as early as 4 dpi.

Apart from PR proteins and proteinase inhibitors, we noticed a significant up-regulation of genes associated with cell death such as lipoxygenase (LOX) [44], cannabidiolic synthase [49], peroxidases [59], alpha-dioxygenase 1-like enzyme [60], ornithine decarboxylase [68], patatin-like phospholipase [71] and non-symbiotic haemoglobin 1 [72]. Among these, LOX was one of the highest up-regulated genes with 9.2 log2-fold change. This gene was 99% identical in sequence to a previously characterized pepper LOX gene which induced cell death phenotype upon transient expression in leaves [44]. Additionally, LOX is a key enzyme in jasmonic acid (JA) biosynthesis suggesting a role in cell signalling [76].

Another up-regulated defence related gene was pleotrophic drug resistant (PDR) protein. According to studies on a previously reported homologue, this gene may effect fungal pathogens through transport of an antifungal compound sclareol [54]. The most highly up-regulated gene (9.46 log2-fold) in the entire dataset was pepper esterase (PepEST), which was 99% identical to *PepEST* of *C*. *annuum*, a gene that is induced during early events of incompatible plant-pathogen interactions. In capsicum, PepEST has been shown to prevent appressorium formation of *Colletotricum gloeosporioides* [77]. However, its mode of action in defence against viral pathogens is not known.

### Cell signalling-related genes

We have also identified genes that may be involved in hormone-mediated signalling pathways and signal transduction induced upon CaCV infection (S3 Table). Plant hormones play a vital role in disease resistance through regulation of expression of gene networks associated with defense responses [78]. Involvement of salicylic acid (SA), JA and ethylene (ET) have been extensively studied in several plant-pathogen interactions and has been covered in many reviews [78–80]. As discussed above, PR proteins are highly represented among up-regulated genes in our data set. Most PR proteins are induced following the activation of signalling molecules such as SA, JA and ET [34]. Expression of PR proteins such as PR-1, -2 and -5 are promoted through increased SA levels, while PR-3, -4 and -12 defensin expression are promoted through JA and ET [34, 80]. One of the significantly up-regulated genes thought to be involved in cell signalling was 1-aminocyclopropane-1-carboxylate oxidase (ACC oxidase). ACC oxidases catalyze oxidative cleavage of ACC to form ET and induces leaf senescence [45]. In addition, ET-responsive transcription factors, ET receptor and sensor genes were also found up-regulated. All these up-regulated genes suggest increased levels of ET production and activity. Another cell-signaling related gene was cytochrome P450, which was 99% identical to a putative cytochrome P450 gene (*CaCYP1*) of chili pepper (*C*. *annuum* L. Bukang). *CaCYP1* is thought to play a defense role through SA and abscisic acid (ABA) signalling pathways [47]. UDP-glucosyltransferase was another SA-associated up-regulated gene which is thought to be involved in the biosynthesis of SA [81]. A previously reported homologue in capsicum was also shown to be involved in tobacco mosaic virus resistance through controlling SA accumulation [57]. Auxin and ABA have also been implemented in plant-pathogen interactions [80]. Auxin has been shown to increase virulence of viral pathogens and thereby increase susceptibility. By contrast, we annotated several genes for auxin-responsive proteins among up-regulated genes in this study. Effects of these genes during disease resistance warrant future research.

In addition to genes involved in hormone-mediated signalling, we found several other highly up-regulated genes which may function as signal molecules such as patatin-like phospholipase and apoplastic invertase [48, 71]. Invertase is a key hydrolytic enzyme in sucrose metabolism and is known to produce a metabolic signal which induces defence-related genes and down-regulates photosynthesis [48]. Another set of up-regulated genes was several genes coding for glutathione-S-transferases (GST), which were classified with ‘auxin-activated signalling pathway’ and ‘protein binding’ GO terms. A previous study reported that different auxins activate and bind to GST and may have different functions [82]. Hence, functions of GST genes in our dataset could not be predicted without further study. We observed a 7.28 log2-fold increase in omega-6 fatty acid desaturase (FAD). Various FADs that are induced during biotic and abiotic stress conditions have been reviewed [52]. In parsley (*Petroselinum crispum* L.) a FAD with high sequence similarity to Omega-6 FAD has been shown to be induced in response to a fungal elicitor and thought to function as signalling molecule in the early defense response [51].

### Secondary metabolism-related genes

Several genes involved in the biosynthesis of secondary metabolic compounds such as antibiotics and phytoalexins were also up-regulated in CaCV-resistant line upon virus infection. Mapping enzymes into KEGG pathways revealed the most enriched pathway was ‘biosynthesis of antibiotics’ with 60 up-regulated enzymes involved in biosynthesis of antibiotics and links with other biosynthetic pathways of defense-related secondary metabolites such as phenylalanine, sesquiterpenoids and mevalonate. In addition, phenylalanine, tyrosine and tryptophan biosynthesis (20 enzymes), phenylalanine metabolism (11 enzymes), phenylpropanoid biosynthesis (10 enzymes) and terpenoid backbone synthesis pathway (9 enzymes) were also found enriched. These pathways lead to the synthesis of secondary metabolites involved in plant defense against pathogens including phytoalexins, phytoanticipins, phenolic compounds such as alkaloids, terpenoids and lectins. One of the compounds synthesized through phenylalanine metabolism was capsaicin. Capsaicin is one of the most abundant capsaicinoids in capsicum [83]. Accumulation of capsaicinoids cause pungency in fruits [84]. Interestingly, these compounds also possess antimicrobial and antifungal properties and induce expression of defense-related genes such as chitinases suggesting potential role in plant defense [85, 86]. Up-regulation of enzymes involved in capsaicin synthesis implies accumulated capsaicin may be involved in defense responses against CaCV. Capsaicin may affect integrity of the virus’ lipid envelope but this is difficult to validate due to fast HR response in the R line. Previously, capsaicin has been shown to interact with phospholipids and alter membrane function [87]. The 5-*epi*-aristolochene synthase (EAS) was the second most highly up-regulated gene in our dataset. EAS catalyzes the production of 5-epi-aristolochene, a precursor of capsidiol, the main phytoalexin produced by capsicum which has been previously shown to be induced by elicitors [43, 88]. We identified two candidate genes involved in biosynthesis of mevalonate, which is the precursor of a class of plant defense metabolites, sesquiterpene phytoalexins. The 3-hydroxy-3-methylglutaryl-CoA synthase (HMGA) and 3-hydroxy-3-methylglutaryl-CoA reductase (HMGR) are key enzymes that regulate mevalonate pathway [89].

### Putative disease resistance genes

The most enriched GO term of the entire dataset was ‘nucleotide binding’ in the MF domain (S3 Table). Annotation showed 214 up-regulated nucleotide binding protein genes. Of those, receptor-like protein kinases and abc transporter family proteins were the most prominent. In addition, endonuclease dicer homolog 2, a component of the RNA silencing defense showed 2.50 log2-fold increased gene expression. Among the other nucleotide binding protein genes, we were most interested in candidate disease resistance genes.

Pathogen resistance mediated through specific disease resistance proteins is often associated with localized cell death at the site of infection and downstream signal transduction for systemic resistance [90]. Majority of known disease resistance genes (Rg) in plants belong to the nucleotide-binding (NB) leucine-rich repeat (LRR) gene family. The NB region is part of a complex NB-ARC domain [91]. Based on the domains present in the N-terminus, disease resistance genes are classified into two groups. Genes containing Toll interleukin-1 receptor (TIR) are referred to as TIR-NB-LRR (TNL) while the other group without TIR is referred to as CNL [92]. Some members of CNL contain a coiled-coil (CC) structure at the N-terminus [93]. In our dataset, we identified 12 putative NB-LRR family disease resistance genes that were up-regulated in R capsicum line. Rg-3 appeared to encode a complete TIR-NB-LRR gene model while other genes were of the CNL type. CNLs were found as partial genes that lacked specific domains and contained only, for example CC-, NB- and CC-NB. Partial NB-LRR genes have been previously reported in genome annotations of *Arabidopsis*, *Brassica rapa* and potato [94–96]. Two genes, Rg-2 and -3 contained more than one transcript sequence per gene. We considered those sequences as transcript isoforms of the same gene. Rg-2 gene contained 4 transcript isoforms with NB- and CC-NB- of varying sizes. One of the Rg-3 transcript isoforms contained complete TIR-NB-LRR gene model while the other transcript isoforms contained NB- and partial LRR and lacked TIR domain. Transcript isoforms may arise due to alternative splicing (AS) events, which are thought to play an important role in regulation of gene expression and are considered as a critical regulatory mechanism in plants to defend against pathogens [97–99]. To determine potential mechanisms of AS events, transcript isoforms were aligned using Geneious software and manually searched for exon-exon splice junctions and transcription start sites (TSS) [100]. TSSs were further confirmed through Tophat aligned files generated during transcript assembly. Transcript isoforms in Rg-2 seemed to be generated through intron retention, alternative splicing and selection of alternative 5’ TSS while in Rg-3, they appear to have occurred through alternative TSSs and an alternative splicing event. Interestingly, alternative splicing in Rg-3 has utilized a non-canonical splice donor/acceptor (GT/CA instead of GT/AG). Whatever the mechanism used, AS appears to lead to truncated proteins. Functional consequences of AS derived transcript isoforms in our dataset remain to be evaluated in future research. Previously characterized AS disease resistance genes such as Arabidopsis *RPS4*, *Medicago tranculata RCT1* and tobacco *N* revealed truncated proteins are essential to exhibit complete resistance, while AS transcript isoforms alone showed partial or no resistance [98, 99, 101]. On the other hand, disease resistance genes such as flax *L6* and tomato *BS4* do not require alternatively spliced transcript isoforms to lead to complete resistance [102, 103].

Of the 12 putative disease resistance genes in our dataset, only 8 mapped to predicted protein-coding regions in the *C*. *annuum* cv. Zunla-1 reference genome. Rg10 expression was limited to the R line but it mapped with 100% sequence identity to a protein-coding region of the *C*. *annuum* reference genome. Therefore, we consider it unlikely that this is the gene responsible for triggering the resistance response to CaCV, which was introgressed from *C*. *chinense*. However, Rg10 is likely involved downstream as part of the response cascade leading to HR in the R line. It is possible that the other genes could not be aligned to predicted protein-coding regions in the capsicum reference genome due to incomplete annotation or novelty of genes that are not present in *C*. *annnum*-Zunla inbred line. We assumed that these four unmapped genes may originate from CaCV-resistant *C*. *chinense*. All four candidate genes contained a single transcript sequence per gene with partial NB-LRR gene models such as Rg-7, NB-, Rg-8, RX-CC-NB-, Rg-9, partial NB- and Rg-11, RX-CC-. To determine whether Rg-7, -8, -9 and -11 genes originated from *C*. *chinense*, transcript sequences were BLAST searched against *C*. *chinense* (v0.5) scaffolds (Pepper Genome Platform). Scaffolds with hits were selected based on E-value and percentage of sequence coverage. Selected scaffolds were aligned with corresponding transcript sequences using Geneious software and manually annotated [100]. We were able to predict NB-LRR gene models for Rg-7 and Rg-8.

Rg-8 transcript sequence contained a complete RX-CC-NB-LRR model and could easily be aligned *to C*. *chinense* scaffold 14663 containing a sequence with 100% identity and coverage, implying that it originated from *C*. *chinense*. Rg-8 protein was encoded by a single open reading frame (ORF) of 3792 nucleotides (nt) without introns. In a subsequent GenBank search Rg-8 protein had 78% identity with 95% sequence coverage to a *C*. *annuum* root-knot nematode resistance protein (accession ACI43068, unpublished) that was not annotated in the cv. Zunla reference genome. Rg-7 contained a partial gene model (NB-) and aligned with several *C*. *chinense* scaffolds (hereafter referred to as candidate scaffolds) with 87–95% identity due to the highly conserved NB domain. To obtain a complete gene model for Rg-7, paired-end Illumina reads were mapped to each candidate scaffold. A complete NB-LRR gene model could be predicted from the sequences aligned to *C*. *chinense* scaffold 35219. However, this gene model lacked CC domain. A consensus sequence of 3870 nt containing a single ORF without introns was derived for Rg-7. Blast searches of the deduced amino acid sequence showed Rg-7 was 82% identical to previously characterized CC-NB-LRR type proteins of *C*. *chinense*, *C*. *chacoense*, *C*. *baccatum*, *C*. *frutescense* and *C*. *annuum* [104]. We could not annotate Rg-9 and -11 transcripts aligned scaffolds and predict gene models.

Since the chromosomal location of CaCV resistance gene in R breeding line is unknown, we searched for possible associations of candidate resistance genes based on genome assemblies. Rg-7 and -8 ORFs were BLAST searched against *C*. *annuum* Zunla-1 and Chiltepin (*C*. *annuum* var. glabriusculum) and hot pepper (*C*. *annuum* cv. CM334) assemblies (Pepper Genome Platform) to determine chromosomal location. BLAST revealed Rg-7 is located on chromosome 11 and Rg-8 on chromosome 6. RNA-Seq and qPCR log2-fold expression of Rg-7 was 3.19 and 4.63 respectively. Fold expression of Rg-8 were less compared to Rg-7, which was 1.64 in RNA-Seq and 1.48 in qPCR. Since a dominant CaCV resistance gene would likely express with higher fold change, it is possible that Rg-7 may be the CaCV resistance gene in R line that has been introgressed from *C*. *chinense*. However, this hypothesis needs to be confirmed experimentally by phenotyping using gene silencing or by transgenic approaches and molecular fine mapping of segregating populations.

### Validation of RNA-Seq gene expression profiles by real-time qPCR

**Fig 3:**
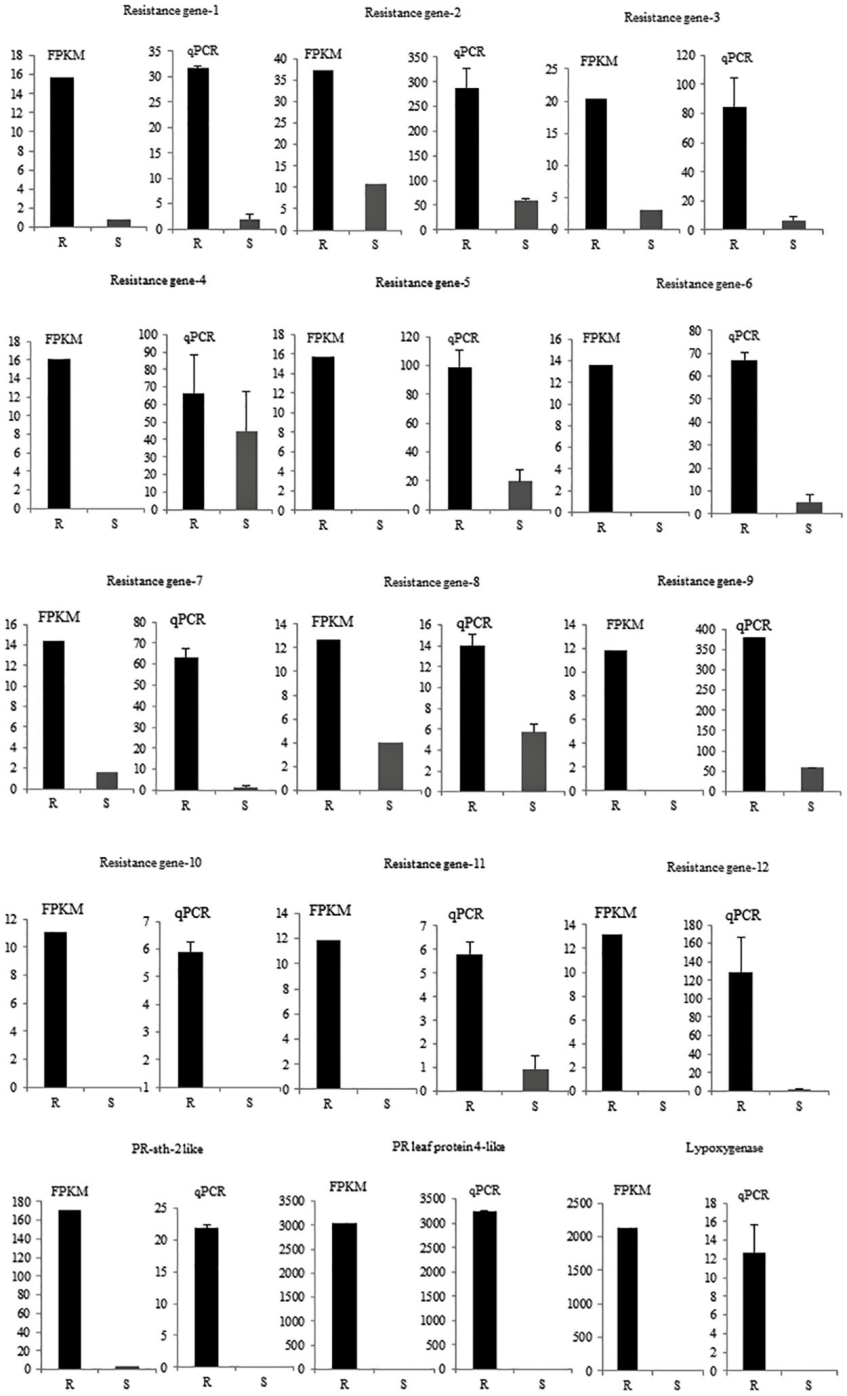
Validation of RNA-Seq gene expression by real-time quantitative PCR (qPCR). FPKM (fragments per kilobase of exon per million fragments mapped) values obtained by RNA-Seq analysis and relative expression levels obtained by qPCR for 15 selected genes in CaCV resistant (R) and susceptible (S) *Capsicum annuum* plants inoculated with capsicum chlorosis virus are shown. Error bars represent the standard error for three biological replicates. Putative resistance genes are consecutively numbered 1–12; PR, pathogenesis-related.

RNA-Seq based gene expression levels of 15 selected genes of interest were validated by real-time qPCR using the same total RNA preparations used for RNA-Seq library preparation. We selected 12 candidate disease resistance genes, two PR protein genes and a lipoxygenase gene to validate gene expression data. Initially, expression patterns of 4 housekeeping genes; actin, TATA box-binding, nuclear cap-binding and GLDH selected from RNA-Seq dataset were tested as potential internal reference genes. Since all these genes showed stable gene expression patterns across all treatments (data not shown), the widely used actin gene was selected as internal reference for qPCR experiments. Expression of target genes was normalized to actin and relative expression levels were calculated for each target gene and compared between R and S lines (Fig 3). Expression levels of disease resistance genes were always less than the highly expressed actin in both R and S lines. Since the calculated qPCR relative expression values were smaller than 1, we multiplied them by a factor of 1000 for plotting data in graphs. Fold changes in target gene expression in R line as compared to S line showed similar trends in qPCR and RNA-Seq (Table 2). However, genes that had zero FPKM values in S line showed a low level of expression in qPCR analysis, indicating higher sensitivity of qPCR and/or insufficient depth of coverage in RNA-Seq.

**Table 2:** Comparison of transcript fold change as detected by RNA-Seq and qPCR.

Transcript Seq. ID	Seq. description	FPKMR line	FPKMS line	log2-fold	qPCR log2-fold^a^	validated
**21345**	Rg1	15.72	0.79	4.29	3.76 ± 0.526	yes
**10552**	Rg2	37.12	10.76	1.78	1.61 ± 0.368	yes
**48569**	Rg3	20.39	2.97	2.77	3.68 ± 0.328	yes
**26003**	Rg4	16.09	0.0	-	0.65 ± 0.201	yes
**26004**	Rg5	15.78	0.0	-	2.3 ± 0.312	yes
**26005**	Rg6	13.67	0.0	-	4.89 ± 0.472	yes
**27596**	Rg7	14.38	1.56	3.19	4.63 ± 0.853	yes
**31761**	Rg8	12.70	4.05	1.64	1.48 ± 0.345	yes
**38700**	Rg9	12.65	0.0	-	2.75 ± 0.209	yes
**40213**	Rg10	11.06	0.0	-	Only in R line	yes
**40214**	Rg11	11.86	0.0	-	2.74 ± 0.671	yes
**40215**	Rg12	13.21	0.0	-	6.81 ± 0.263	yes
**18520**	PR sth-2-like	170.77	3.42	5.63	8.59 ± 0.364	yes
**39531**	PR leaf protein 4-like	3045.13	5.85	9.02	11.76 ± 0.206	yes
**22353**	LOX	2132.67	3.02	9.46	11.30 ± 0.081	yes

^a^qPCR fold changes represent mean of three biological replicates.

## Conclusions

CaCV-induced resistance response was evaluated in a comparative transcriptome analysis of CaCV-resistant and susceptible *C*. *annuum* using Illumina HiSeq sequencing platform and subsequent bioinformatics analysis. CaCV induced strong HR on inoculated leaves of the resistant line as early as 4 days after CaCV infection. We identified a range of defense-related genes involved in this HR and genes that may induce resistance in systemic tissues such as signalling molecules. Genes involved in localized cell death, cell signalling, synthesis of antimicrobial compounds and PR proteins were found highly up-regulated. Two potential CaCV resistance candidate genes were identified in the resistant *C*. *annuum x C*. *chinense* breeding line. These genes were manually annotated in *C*. *chinense* genome with a NB-LRR gene model, which belongs to CNL type resistance genes.

## Supporting Information

S1 Table Sequences of oligonucleotide primers used in qPCR. (DOCX)

S2 Table Statistics of predicted-protein coding genes of capsicum genomes and transcriptomes in comparison to Bell capsicum transcriptome sequenced in this study. (DOCX)

S3 Table Differentially expressed putative resistance response-related genes and respective gene ontology (GO) assignments in response to CaCV infection in CaCV-resistant *Capsicum annuum* in comparison to CaCV-susceptible *C*. *annuum* after 4 days of CaCV inoculation. GO assignments for all transcript variants per gene is indicated. C: Cellular Component, F: Molecular Function, P: Biological Process.
(XLSX)
