# Supplementary material for: Transcriptome Analysis of Capsicum Chlorosis Virus-Induced Hypersensitive Resistance Response in Bell Capsicum
Source: PLoS One. 2016 Jul 11;11(7):e0159085. doi: 10.1371/journal.pone.0159085 (PMC4939944; doi:10.1371/journal.pone.0159085)
Supplement: S1 Table — (DOCX) [file pone.0159085.s001.docx]

**Table S1. Sequences of oligonucleotide primers used in qPCR**

| **Name** | **Forward (5’ to 3’)** | **Reverse (5’ to 3’)** | **Amplicon size (bp)** |
| --- | --- | --- | --- |
| **Actin** | GCTGGACGTGACCTAACTG | GCAGTTTCAAGCTCCTGCTC | 150 |
| **TATA box-binding** | ATGGCGGATCAGGCATTAG | CAGGTCCAACTTGCAGTCC | 120 |
| **nuclear cap-binding** | TGAGCTGTTCTCTCGTGCTG | AGTCTTCAGCATCGTCTCTGG | 119 |
| **GLDH** | CTCGGCAGAGAAAGCAAATC | TGTCCTTGGGAACCTCAATC | 120 |
| **LOX** | TGGAGGGTCCAAGCATTTAG | CTGCTGCCTTTGATGGTTC | 194 |
| **PR-4-like** | ATGCAGGCTTGGCATCTC | TCAGACACCCACAACTCCAC | 145 |
| **PR sth-2-like** | TTCTGCAGATGGAGGATGTG | GCAGAAGGATTGGTGAGGAG | 153 |
| **Rg1** | TACGATGGATGAGAGCTGGA | GATCTCAAGCCACGAAGAGG | 181 |
| **Rg2** | GTCGGTATTGGGTCAGTTGG | ACCTGAAGCATGCCAAGAAG | 154 |
| **Rg3** | TTTCACGACCTCCATCTTCC | CCCAGCTGGTAAACACTCTTG | 147 |
| **Rg4** | CAGAAGGGCCAAGAAATCAG | TTGCCTAATCCACCCATACC | 193 |
| **Rg5** | TGGCAGAGAGAAGCATGTGA | TCCTACGAAAGAGGTCCCAAC | 175 |
| **Rg6** | GCGGAAGGATTCATACCAAG | ATGGCAAGATCCCGAAGTAG | 158 |
| **Rg7** | CTGTCTGATGAAGCCTCTTGG | TCTCTCCACTCGTCCACCTC | 185 |
| **Rg8** | ATGAAAGCCACGGACTCATC | ATTCGAGGGCTTCTTTCACC | 160 |
| **Rg9** | GCGTGCACTCGTTCTCATT | GGCATCCCATGCTTCACT | 124 |
| **Rg10** | TGGCGAGAGCCAAGTTATG | ACACCGAAGACATGGAGGAC | 183 |
| **Rg11** | TGGAACCTAGGAAACCCACA | AGGCGTAAGGGCATAGTTGA | 172 |
| **Rg12** | TCACTTGCCACAGCACCTAC | CCTCTGCTGCCCATAATCTC | 104 |

GLDH; galactono-lacton dehydrogenase, LOX; lipoxygenase, PR; pathogenesis related protein, Rg 1-12; disease resistance gene 1-12
