# Supplementary material for: Transcriptome Analysis of Capsicum Chlorosis Virus-Induced Hypersensitive Resistance Response in Bell Capsicum
Source: PLoS One. 2016 Jul 11;11(7):e0159085. doi: 10.1371/journal.pone.0159085 (PMC4939944; doi:10.1371/journal.pone.0159085)
Supplement: S2 Table — (DOCX) [file pone.0159085.s002.docx]

**Table S2. Statistics of predicted-protein coding genes of capsicum genomes and transcriptomes in comparison to Bell capsicum transcriptome sequenced in this study**

| **Source** | **No. of protein-coding genes** | **Reference** |
| --- | --- | --- |
| **Genome** |  |  |
| *C. annuum* Zunla-1 | 35,336 | [[1](#_ENREF_1)] |
| *C. annuum* var*.* glabriusculum-Chiltepin | 34,476 | [[1](#_ENREF_1)] |
| *Capsicum annuum* cv. CM334 | 34,903 | [[2](#_ENREF_2)] |
| **Transcriptome** |  |  |
| *C. frutescens* (chili pepper) | 40,479 | [[3](#_ENREF_3)] |
| *C. annuum* (Bell capsicum) | 34,905-34,986 | this study |

References

1. Qin C, Yu C, Shen Y, Fang X, Chen L, et al. (2014) Whole-genome sequencing of cultivated and wild peppers provides insights into Capsicum domestication and specialization. Proceedings of the National Academy of Sciences 111: 5135-5140.

2. Kim S, Park M, Yeom S-I, Kim Y-M, Lee JM, et al. (2014) Genome sequence of the hot pepper provides insights into the evolution of pungency in Capsicum species. Nature Genetics 46: 270-278.

3. Liu S, Li W, Wu Y, Chen C, Lei J (2013) De novo transcriptome assembly in chili pepper (Capsicum frutescens) to identify genes involved in the biosynthesis of capsaicinoids. PloS one 8: e48156.
